# Validation of 58 autosomal individual identification SNPs in three Chinese populations

**DOI:** 10.3325/cmj.2014.55.10

**Published:** 2014-02

**Authors:** Yi-Liang Wei, Cui-Jiao Qin, Hai-Bo Liu, Jing Jia, Lan Hu, Cai-Xia Li

**Affiliations:** 1 Key Laboratory of Forensic Genetics, Institute of Forensic Science, Ministry of Public Security, Beijing, P.R. China; 2 Key Laboratory of Medical Epigenetics, Tianjin Research Center of Basic Medical Sciences, Tianjin, P.R. China; 3 Institution of Forensic Science of Bingtuan Public Security Bureau, Xinjiang, P.R. China; 4 Faculty of Basic Medical Sciences, Chongqing Medical University, Chongqing, P.R. China

## Abstract

**Aim:**

To genotype and evaluate a panel of single-nucleotide polymorphisms for individual identification (IISNPs) in three Chinese populations: Chinese Han, Uyghur, and Tibetan.

**Methods:**

Two previously identified panels of IISNPs, 86 unlinked IISNPs and SNP*for*ID 52-plex markers, were pooled and analyzed. Four SNPs were included in both panels. In total, 132 SNPs were typed on Sequenom MassARRAY^®^ platform in 330 individuals from Han Chinese, Uyghur, and Tibetan populations. Population genetic indices and forensic parameters were determined for all studied markers.

**Results:**

No significant deviation from Hardy-Weinberg equilibrium was observed for any of the SNPs in 3 populations. Expected heterozygosity (H_e_) ranged from 0.144 to 0.500 in Han Chinese, from 0.197 to 0.500 in Uyghur, and from 0.018 to 0.500 in Tibetan population. Wright's F_st_ values ranged from 0.0001 to 0.1613. Pairwise linkage disequilibrium (LD) calculations for all 132 SNPs showed no significant LD across the populations (r^2^<0.147). A subset of 58 unlinked IISNPs (r^2^<0.094) with H_e_>0.450 and F_st_ values from 0.0002 to 0.0536 gave match probabilities of 10^−25^ and a cumulative probability of exclusion of 0.999992.

**Conclusion:**

The 58 unlinked IISNPs with high heterozygosity have low allele frequency variation among 3 Chinese populations, which makes them excellent candidates for the development of multiplex assays for individual identification and paternity testing.

Single-nucleotide polymorphisms (SNPs) are often used as a supplementary tool to short tandem repeats (STRs) analysis (1) since they show advantages over STRs in degraded DNA detection (2), kinship analysis (3), ancestry inference (4,5), and physical traits analysis (6). Different SNPs groups have been selected according to defined purposes (4,5,7,8).

In the last decades, several panels for individual identification have been developed (2,9-11). Kidd et al (12) defined an ideal SNP panel for individual identification (IISNP) as a group of statistically independent SNPs that showed little frequency variation among different populations with high heterozygosity. Based on this criterion, Pakstis et al selected 86 unlinked candidate individual identification SNPs (IISNPs) with average heterozygosity >0.4 and F_st_ values <0.06 for 44 major populations across the world (1). However, the sample sizes for Chinese populations were very limited. On the other hand, the SNP*for*ID consortium (*www.snpforid.org*) developed a 52-plex SNPs assay for individual identification (7). This assay was validated by Břrsting et al (13-15) according to the ISO 17025 standard and used for routine casework. This assay worked well in several European countries but showed a somewhat larger frequency variation among populations from other continents (16-18).

In order to collect an ideal SNP panel for individual identification and evaluate its performance in Chinese populations, we pooled the previous 86 IISNPs and 52-plex SNPs together, and typed them in 330 samples of three Chinese population groups: Han, Tibetan, and Uyghur.

## Material and methods

### Sample collection and DNA preparation

Population samples included 110 Han Chinese recruited from Beijing, 110 Uyghurs from Urumqi, and 110 Tibetans from Lhasa. Patients whose names were obtained from general practitioners’ registers were invited to participate in the study. They were asked to complete a brief general information questionnaire including the data about name, place, sex, age, and ethnic and racial information about the last three generations of their family. Volunteers whose ancestry information met our research requirements were recruited. In total, 330 whole blood samples of 1 mL were obtained from healthy volunteers (176 men and 154 women) who had provided written informed consent. Ethical approval was received from the Review Board, Institute of Forensic Sciences, Ministry of Public Security.

DNA was isolated and purified with a QIAamp^®^ DNA blood midi kit (QIAGEN, Hilden, Germany). DNA quantification was performed with adding 1.5 μL DNA samples in solution on NanoDrop^®^ ND-1000 spectrophotometer (Thermo Scientific, Wilmington, DE, USA).

### SNP typing

SNPs genotyping was performed using the Sequenom MassARRAY^®^ platform with the iPLEX GOLD chemistry (Sequenom, San Diego, CA, USA) following the manufacturer’s protocols. Polymerase chain reaction (PCR) primers and locus-specific extension primers were designed using MassARRAY^®^ Assay Design software package (v. 3.1). DNA template of 50 ng was used in each multiplexed PCR well. PCR products were treated with shrimp alkaline phosphatase (USB, Cleveland, OH, USA) before the iPLEX GOLD primer extension reaction. The single base extension products were desalted with SpectroCLEAN^®^ resin (Sequenom), and then an aliquot of 10 nL of the desalted product was spotted onto a 384-format SpectroCHIP^®^ with the MassARRAY^®^ Nanodispenser. Mass determination was done with the MALDI-TOF mass spectrometer. The MassARRAY^®^ Typer 4.0 software was employed for data acquisition.

### Statistical analysis

Population indices including allele and genotype frequency, expected heterozygosity (H_e_), Hardy-Weinberg equilibrium (HWE), and Wright's F_st_ value, were calculated for each SNP marker with Genepop v4.2. F_st_ and δ values were used to measure variance in allele frequencies among populations. Random match probability (RMP), probability of exclusion (PE), and other forensic parameters were calculated with PowerStats v. 12. HaploView v. 4.2 genetics software was implemented to estimate linkage disequilibrium (LD) values (r^2^) of each pair of SNPs. HapMap genotype data were used for population comparison analyses. All individual and marker data were filtered with more than 20% of missing alleles.

## Results

We typed 132 SNPs in 330 individuals from Han, Uyghur, and Tibetan subpopulations (Supplementary Table 1)(supplementary table 1). Four SNPs were included in both panels and 2 were excluded due to the failure of multiplex detection. HWE tests demonstrated no significant deviation from expected values (*P* < 0.001, after Bonferroni correction for multiple testing). There were no significant LD values among the SNPs (pair-wise r^2^<0.147). H_e_ ranged from 0.144 to 0.500 in Han, from 0.197 to 0.500 in Uyghur, and from 0.018 to 0.500 in Tibetan population. Wright's F_st_ values were from 0.0001 to 0.1613. Allele frequencies ranged from 0.114 to 0.922 in Han, from 0.118 to 0.889 in Uyghur, and from 0.055 to 0.991 in Tibetan population. Pair-wise δ values ranged from 0 to 0.324 (Supplementary Table 2)(supplementary table 2).

Among the 132 IISNPs, we identified a set of 58 unlinked markers (r^2^<0.094) separated on 22 autosomal chromosomes. This subset consisted of 41 markers from the Pakstis' panel (20 recommended ones), 14 markers from the SNP*for*ID panel, and 3 markers from both panels. H_e_ values were >0.450 and F_st_ values ranged from 0.0002 to 0.0536. Match probabilities of Han, Uyghur, and Tibetan populations were 5.91 × 10^−25^, 7.79 × 10^−25^, and 6.61 × 10^−25^, respectively. The cumulative probability of exclusion for these three populations was 0.999992.

## Discussion

Each of the 58 unlinked markers from two IISNP panels had high heterozygosity (H_e_>0.450) and very similar frequencies (F_st_<0.060) across the three Chinese population samples. One hundred and thirty-two autosomal IISNPs were screened in three Chinese populations. These markers have previously been tested in many populations (1,8,16,18,19). In our study, several markers displayed wide frequency variation among the three Chinese populations, such as rs1355366, rs717302, rs1886510, and rs1493232. These markers were not suitable for individual identification. An ideal panel of IISNPs should include more than 50 statistically independent markers (low LD values), each with high heterozygosity (approaching 0.5) and low F_st_ values (approaching 0). The panel meeting this criterion should have high power of discrimination and allow for universal uses of individual identification. Thus, we considered the subset of 58 unlinked SNPs, which had previously been tested for a large number of world-wide populations, an excellent panel for practical use in Chinese populations. It had match probabilities of 10^−25^, which is comparable to the CODIS STR panel and other existing SNP typing systems (7,8,19). As we know, in the field of forensic science SNPs are now studied with much more interest than STRs. However, it is not enough just to develop a SNP panel capable of individual identification for a new marker to be widely accepted in routine detection. SNPs panels of the Y (20,21) chromosomal and mtDNA (22,23) should also be studied and developed in terms of requirements for paternal and maternal lineage searches of individuals.

Individual identification requires an easy-to-use multiplex SNP genotyping system, which can be read with capillary electrophoresis. Furthermore, a stable and reliable detection system should include more population samples. In conclusion, the 58 unlinked IISNPs identified here would be an ideal panel for further multiplex system optimization and validation among Chinese populations.
