# Supplementary material for: Validation of 58 autosomal individual identification SNPs in three Chinese populations
Source: Croat Med J. 2014 Feb;55(1):10–3. doi: 10.3325/cmj.2014.55.10 (PMC3944413; doi:10.3325/cmj.2014.55.10)
Supplement: Supplementary Table 1 [file CroatMedJ_55_s001.pdf]

Table S1. Wright's F_{st}, expected heterozygosity (H_e), random match probability (RMP), and probability of exclusion (PE) of 132 single nucleotide polymorphisms for individual identification (IISNP)

No.	The 58 unlinked IISNPs [*]	The 44 IISNPs recommended by Pakstis et al [†]	dbSNP rs#	Chromesome	Nucleotide Position on Map Build 36.2	Source	F_{st} (3 Pop.) [‡]	H_e			RMP			PE		
								Han	Uyghur	Tibetan	Han	Uyghur	Tibetan	Han	Uyghur	Tibetan
1	√		rs1490413	1	4367323	Pakstis et al & SNP*for*ID	0.0157	0.497	0.499	0.470	0.376	0.375	0.391	0.187	0.187	0.180
2		√	rs7520386	1	14155402	Pakstis et al	0.0023	0.299	0.375	0.325	0.535	0.461	0.509	0.127	0.152	0.136
3			rs4847034	1	105717631	Pakstis et al	0.0459	0.499	0.426	0.498	0.376	0.420	0.376	0.187	0.168	0.187
4		√	rs560681	1	160786670	Pakstis et al	0.0297	0.438	0.494	0.500	0.412	0.378	0.375	0.171	0.186	0.187
5		√	rs1294331	1	233448413	Pakstis et al	0.0095	0.401	0.474	0.460	0.439	0.389	0.398	0.160	0.181	0.177
6	√		rs10495407	1	238439308	SNP*for*ID	0.0047	0.454	0.452	0.458	0.401	0.402	0.399	0.176	0.175	0.176
7	√		rs891700	1	239881926	Pakstis et al & SNP*for*ID	0.0034	0.499	0.498	0.500	0.376	0.376	0.375	0.187	0.187	0.188
8	√		rs1413212	1	242806797	SNP*for*ID	0.0103	0.493	0.495	0.495	0.379	0.378	0.378	0.186	0.186	0.186
9			rs876724	2	114974	SNP*for*ID	0.0230	0.495	0.446	0.416	0.378	0.407	0.427	0.186	0.173	0.165
10	√	√	rs1109037	2	10085722	Pakstis et al	0.0067	0.480	0.487	0.500	0.386	0.382	0.375	0.182	0.184	0.188
11	√	√	rs993934	2	124109213	Pakstis et al	0.0006	0.490	0.500	0.498	0.380	0.375	0.376	0.185	0.187	0.187
12		√	rs12997453	2	182413259	Pakstis et al	0.0017	0.439	0.411	0.446	0.411	0.431	0.407	0.171	0.163	0.173
13	√		rs907100	2	239563579	SNP*for*ID	0.0023	0.486	0.500	0.494	0.382	0.375	0.378	0.184	0.188	0.186
14			rs1357617	3	961782	SNP*for*ID	0.0181	0.263	0.300	0.146	0.577	0.535	0.740	0.114	0.128	0.068
15	√	√	rs4364205	3	32417644	Pakstis et al	0.0039	0.476	0.466	0.469	0.388	0.393	0.392	0.181	0.179	0.179
16			rs9866013	3	59488340	Pakstis et al	0.0108	0.364	0.455	0.420	0.471	0.401	0.425	0.149	0.176	0.166
17		√	rs2399332	3	110301126	Pakstis et al	0.0136	0.368	0.466	0.413	0.467	0.393	0.430	0.150	0.179	0.164
18	√		rs1872575	3	113804979	Pakstis et al	0.0046	0.499	0.500	0.500	0.376	0.375	0.375	0.187	0.188	0.187
19			rs1355366	3	190806108	SNP*for*ID	0.1038	0.219	0.474	0.280	0.634	0.389	0.558	0.097	0.181	0.120
20		√	rs6444724	3	193207380	Pakstis et al	0.0073	0.442	0.455	0.380	0.409	0.401	0.457	0.172	0.176	0.154
21	√		rs2046361	4	10969059	Pakstis et al & SNP*for*ID	0.0045	0.476	0.466	0.474	0.388	0.393	0.389	0.181	0.179	0.181
22	√	√	rs279844	4	46329655	Pakstis et al	0.0169	0.488	0.491	0.500	0.381	0.380	0.375	0.184	0.185	0.187
23	√		rs13134862	4	76425896	Pakstis et al	0.0063	0.493	0.497	0.497	0.379	0.377	0.376	0.186	0.187	0.187
24	√		rs1554472	4	157489906	Pakstis et al	0.0074	0.500	0.476	0.478	0.375	0.388	0.387	0.187	0.181	0.182
25		√	rs6811238	4	169663615	Pakstis et al	0.0196	0.465	0.496	0.424	0.395	0.377	0.422	0.178	0.186	0.167
26	√		rs1979255	4	190318080	SNP*for*ID	0.0088	0.483	0.474	0.500	0.384	0.389	0.375	0.183	0.181	0.187
27			rs717302	5	2879395	SNP*for*ID	0.1035	0.167	0.424	0.165	0.708	0.422	0.710	0.076	0.167	0.076
28	√	√	rs159606	5	17374898	Pakstis et al	0.0037	0.499	0.485	0.480	0.375	0.383	0.386	0.187	0.184	0.182
29	√	√	rs13182883	5	136633338	Pakstis et al	0.0141	0.500	0.464	0.486	0.375	0.395	0.382	0.188	0.178	0.184
30			rs7704770	5	159487953	Pakstis et al	0.0142	0.481	0.468	0.401	0.385	0.392	0.439	0.183	0.179	0.160
31			rs315791	5	169735920	Pakstis et al	0.0743	0.373	0.499	0.370	0.463	0.376	0.465	0.152	0.187	0.151

32			rs251934	5	174778678	SNP*for*ID	0.0429	0.256	0.353	0.135	0.585	0.480	0.758	0.112	0.146	0.063
33	√	√	rs338882	5	178690725	Pakstis et al	0.0018	0.477	0.496	0.470	0.387	0.377	0.391	0.182	0.186	0.180
34	√		rs1029047	6	1135939	SNP*for*ID	0.0039	0.465	0.454	0.454	0.395	0.401	0.401	0.178	0.176	0.176
35		√	rs13218440	6	12059954	Pakstis et al	0.0399	0.492	0.463	0.361	0.379	0.396	0.473	0.185	0.178	0.148
36	√		rs2811231	6	55155704	Pakstis et al	0.0432	0.488	0.483	0.472	0.381	0.384	0.390	0.184	0.183	0.180
37	√	√	rs1336071	6	94537255	Pakstis et al	0.0013	0.495	0.498	0.479	0.378	0.376	0.386	0.186	0.187	0.182
38	√		rs1478829	6	120560694	Pakstis et al	0.0010	0.490	0.467	0.471	0.380	0.393	0.391	0.185	0.179	0.180
39	√		rs1358856	6	123894978	Pakstis et al	0.0002	0.499	0.499	0.497	0.375	0.376	0.377	0.187	0.187	0.187
40	√		rs2503107	6	127463376	Pakstis et al	0.0045	0.479	0.498	0.500	0.386	0.376	0.375	0.182	0.187	0.187
41	√		rs2272998	6	148761456	Pakstis et al	0.0045	0.497	0.494	0.496	0.376	0.378	0.377	0.187	0.186	0.186
42		√	rs214955	6	152697706	Pakstis et al	0.0090	0.483	0.496	0.449	0.384	0.377	0.404	0.183	0.186	0.174
43			rs727811	6	165045334	SNP*for*ID	0.0045	0.238	0.197	0.225	0.610	0.665	0.627	0.105	0.089	0.100
44		√	rs6955448	7	4310365	Pakstis et al	0.0020	0.405	0.460	0.442	0.436	0.397	0.409	0.162	0.177	0.172
45			rs917118	7	4457003	SNP*for*ID	0.0090	0.448	0.454	0.375	0.405	0.401	0.461	0.174	0.176	0.152
46			rs1019029	7	13894276	Pakstis et al	0.0548	0.487	0.492	0.437	0.382	0.379	0.412	0.184	0.185	0.171
47	√	√	rs321198	7	137029838	Pakstis et al	0.0082	0.498	0.498	0.466	0.376	0.376	0.393	0.187	0.187	0.179
48			rs737681	7	155990813	SNP*for*ID	0.0288	0.226	0.393	0.375	0.625	0.446	0.461	0.100	0.158	0.152
49			rs763869	8	1375610	SNP*for*ID	0.0343	0.465	0.389	0.309	0.395	0.450	0.525	0.178	0.156	0.131
50		√	rs10092491	8	28411072	Pakstis et al	0.0070	0.446	0.442	0.373	0.407	0.409	0.463	0.173	0.172	0.152
51	√		rs4288409	8	136839229	Pakstis et al	0.0018	0.476	0.451	0.474	0.388	0.403	0.389	0.181	0.174	0.181
52			rs2056277	8	139399116	SNP*for*ID	0.0012	0.205	0.256	0.282	0.653	0.585	0.555	0.092	0.112	0.121
53		√	rs4606077	8	144656754	Pakstis et al	0.0183	0.364	0.452	0.347	0.471	0.402	0.487	0.149	0.175	0.143
54	√		rs1015250	9	1823774	SNP*for*ID	0.0291	0.492	0.472	0.496	0.379	0.390	0.377	0.185	0.180	0.186
55			rs2270529	9	14747133	Pakstis et al	0.0252	0.343	0.436	0.291	0.490	0.413	0.544	0.142	0.170	0.125
56	√	√	rs7041158	9	27985938	Pakstis et al	0.0022	0.470	0.483	0.465	0.391	0.384	0.394	0.180	0.183	0.179
57	√		rs1463729	9	126881448	SNP*for*ID	0.0016	0.492	0.494	0.468	0.379	0.378	0.393	0.185	0.186	0.179
58			rs1360288	9	128968063	SNP*for*ID	0.0054	0.448	0.472	0.408	0.405	0.390	0.433	0.174	0.180	0.163
59		√	rs10776839	9	137417308	Pakstis et al	0.0075	0.480	0.488	0.436	0.386	0.381	0.413	0.182	0.184	0.170
60			rs826472	10	2406631	SNP*for*ID	0.0048	0.280	0.353	0.370	0.558	0.480	0.465	0.120	0.146	0.151
61			rs735155	10	3374178	SNP*for*ID	0.0455	0.308	0.478	0.408	0.527	0.387	0.433	0.130	0.182	0.163
62	√	√	rs3780962	10	17193346	Pakstis et al	0.0211	0.478	0.495	0.493	0.387	0.378	0.379	0.182	0.186	0.186
63			rs1410059	10	97172595	Pakstis et al	0.0209	0.444	0.493	0.500	0.407	0.379	0.375	0.173	0.186	0.187
64		√	rs740598	10	118506899	Pakstis et al	0.0030	0.474	0.470	0.427	0.389	0.391	0.419	0.181	0.180	0.168
65			rs964681	10	132698419	SNP*for*ID	0.0044	0.448	0.440	0.483	0.405	0.410	0.384	0.174	0.172	0.183
66			rs10500617	11	5099393	Pakstis et al	0.0052	0.475	0.433	0.483	0.388	0.415	0.384	0.181	0.170	0.183
67	√	√	rs1498553	11	5709028	Pakstis et al	0.0019	0.500	0.492	0.498	0.375	0.379	0.376	0.187	0.185	0.187
68			rs901398	11	11096221	Pakstis et al & SNP*for*ID	0.0001	0.384	0.366	0.420	0.453	0.469	0.425	0.155	0.149	0.166
69	√		rs6591147	11	105912984	Pakstis et al	0.0012	0.495	0.492	0.478	0.378	0.379	0.387	0.186	0.185	0.182
70		√	rs10488710	11	115207176	Pakstis et al	0.0032	0.411	0.446	0.380	0.431	0.406	0.457	0.163	0.173	0.154

71			rs590162	11	122195989	Pakstis et al	0.0513	0.439	0.499	0.440	0.411	0.376	0.410	0.171	0.187	0.172
72			rs2076848	11	134667546	SNP*for*ID	0.0357	0.448	0.500	0.444	0.405	0.375	0.408	0.174	0.187	0.173
73			rs2107612	12	888320	SNP*for*ID	0.0326	0.217	0.336	0.151	0.637	0.497	0.733	0.097	0.140	0.069
74			rs2255301	12	6909442	Pakstis et al	0.0074	0.439	0.491	0.468	0.411	0.380	0.393	0.171	0.185	0.179
75	√	√	rs2269355	12	6945914	Pakstis et al	0.0038	0.495	0.497	0.489	0.378	0.377	0.380	0.186	0.187	0.185
76	√		rs2111980	12	106328254	SNP*for*ID	0.0035	0.486	0.489	0.453	0.382	0.381	0.402	0.184	0.185	0.175
77		√	rs10773760	12	130761696	Pakstis et al	0.0001	0.444	0.478	0.474	0.407	0.387	0.389	0.173	0.182	0.181
78			rs1335873	13	20901724	SNP*for*ID	0.0134	0.395	0.476	0.418	0.444	0.388	0.426	0.158	0.181	0.165
79			rs1886510	13	22374700	SNP*for*ID	0.1613	0.248	0.429	0.018	0.596	0.418	0.964	0.109	0.169	0.009
80	√	√	rs1058083	13	100038233	Pakstis et al	0.0005	0.451	0.480	0.483	0.403	0.386	0.384	0.174	0.182	0.183
81	√		rs354439	13	106938411	SNP*for*ID	0.0025	0.492	0.499	0.498	0.379	0.376	0.376	0.185	0.187	0.187
82	√		rs1454361	14	25850832	SNP*for*ID	0.0065	0.497	0.491	0.460	0.377	0.380	0.397	0.187	0.185	0.177
83	√	√	rs722290	14	53216723	Pakstis et al	0.0029	0.498	0.497	0.491	0.376	0.377	0.380	0.187	0.187	0.185
84			rs873196	14	98845531	SNP*for*ID	0.0414	0.263	0.401	0.208	0.577	0.439	0.648	0.114	0.160	0.093
85		√	rs4530059	14	104769149	Pakstis et al	0.0020	0.407	0.398	0.334	0.435	0.442	0.499	0.162	0.159	0.139
86		√	rs1821380	15	39313402	Pakstis et al	0.0241	0.432	0.449	0.498	0.416	0.404	0.376	0.169	0.174	0.187
87	√		rs8037429	15	53616909	SNP*for*ID	0.0053	0.493	0.500	0.483	0.379	0.375	0.384	0.186	0.187	0.183
88	√		rs1528460	15	55210705	SNP*for*ID	0.0368	0.485	0.469	0.490	0.383	0.392	0.380	0.184	0.180	0.185
89			rs729172	16	5606197	SNP*for*ID	0.0609	0.202	0.431	0.282	0.658	0.417	0.555	0.091	0.169	0.121
90		√	rs2342747	16	5868700	Pakstis et al	0.0154	0.426	0.313	0.421	0.420	0.521	0.424	0.168	0.132	0.166
91	√		rs7205345	16	7520254	Pakstis et al	0.0106	0.455	0.496	0.461	0.401	0.377	0.397	0.176	0.187	0.177
92	√	√	rs430046	16	78017051	Pakstis et al	0.0019	0.464	0.464	0.483	0.395	0.395	0.384	0.178	0.178	0.183
93			rs1382387	16	80106361	SNP*for*ID	0.0017	0.442	0.403	0.416	0.410	0.438	0.427	0.172	0.161	0.165
94		√	rs9905977	17	2919393	Pakstis et al	0.0140	0.473	0.436	0.496	0.389	0.413	0.377	0.181	0.170	0.186
95			rs740910	17	5706623	SNP*for*ID	0.0381	0.144	0.274	0.086	0.743	0.565	0.838	0.067	0.118	0.042
96	√		rs4796362	17	6811529	Pakstis et al	0.0006	0.498	0.497	0.500	0.376	0.377	0.375	0.187	0.187	0.187
97			rs8070085	17	41341984	Pakstis et al	0.0165	0.422	0.431	0.489	0.423	0.417	0.380	0.167	0.169	0.185
98	√		rs1004357	17	41691526	Pakstis et al	0.0026	0.486	0.451	0.485	0.382	0.403	0.383	0.184	0.175	0.184
99			rs1027895	17	46510697	Pakstis et al	0.0049	0.472	0.448	0.413	0.390	0.405	0.430	0.180	0.174	0.164
100			rs938283	17	77468498	SNP*for*ID	0.0174	0.247	0.208	0.104	0.599	0.648	0.810	0.108	0.093	0.049
101		√	rs8078417	17	80461935	Pakstis et al	0.0076	0.461	0.384	0.431	0.397	0.453	0.417	0.177	0.155	0.169
102	√		rs2291395	17	80526139	Pakstis et al	0.0006	0.482	0.497	0.487	0.385	0.376	0.382	0.183	0.187	0.184
103	√		rs3744163	17	80739859	Pakstis et al	0.0136	0.496	0.489	0.494	0.377	0.381	0.378	0.186	0.185	0.186
104			rs9546538	17	84456735	Pakstis et al	0.0091	0.469	0.431	0.489	0.392	0.417	0.380	0.179	0.169	0.185
105			rs1493232	18	1127986	SNP*for*ID	0.1356	0.393	0.483	0.397	0.446	0.384	0.443	0.158	0.183	0.159
106	√	√	rs9951171	18	9749879	Pakstis et al	0.0073	0.483	0.499	0.500	0.384	0.375	0.375	0.183	0.187	0.187

107	√		rs7229946	18	22739001	Pakstis et al	0.0026	0.464	0.483	0.482	0.395	0.384	0.385	0.178	0.183	0.183
108	√		rs985492	18	29311034	Pakstis et al	0.0020	0.495	0.483	0.496	0.378	0.384	0.377	0.186	0.183	0.186
109	√		rs521861	18	47371014	Pakstis et al	0.0043	0.492	0.500	0.485	0.379	0.375	0.383	0.185	0.188	0.184
110	√	√	rs1736442	18	55225777	Pakstis et al	0.0043	0.460	0.468	0.464	0.398	0.393	0.395	0.177	0.179	0.178
111			rs1024116	18	75432386	SNP*for*ID	0.0683	0.231	0.437	0.235	0.618	0.412	0.612	0.102	0.171	0.104
112			rs719366	19	28463337	SNP*for*ID	0.0287	0.340	0.474	0.434	0.494	0.389	0.415	0.141	0.181	0.170
113	√	√	rs576261	19	39559807	Pakstis et al	0.0003	0.488	0.496	0.500	0.382	0.377	0.375	0.184	0.186	0.188
114			rs1031825	20	4447483	SNP*for*ID	0.0145	0.436	0.452	0.495	0.413	0.402	0.378	0.170	0.175	0.186
115		√	rs445251	20	15124933	Pakstis et al	0.0001	0.442	0.460	0.420	0.409	0.397	0.425	0.172	0.177	0.166
116			rs12480506	20	16241416	Pakstis et al	0.0265	0.465	0.382	0.489	0.394	0.455	0.381	0.178	0.155	0.185
117	√		rs2567608	20	23017082	Pakstis et al	0.0043	0.479	0.484	0.481	0.386	0.384	0.385	0.182	0.183	0.183
118			rs1005533	20	39487110	SNP*for*ID	0.0034	0.442	0.451	0.464	0.410	0.403	0.395	0.172	0.174	0.178
119		√	rs1523537	20	51296162	Pakstis et al	0.0280	0.471	0.500	0.433	0.391	0.375	0.415	0.180	0.187	0.170
120	√		rs722098	21	16685598	SNP*for*ID	0.0441	0.497	0.480	0.472	0.376	0.386	0.390	0.187	0.182	0.180
121			rs464663	21	28023370	Pakstis et al	0.0223	0.390	0.488	0.464	0.448	0.381	0.395	0.157	0.184	0.178
122			rs2830795	21	28608163	SNP*for*ID	0.0450	0.500	0.422	0.460	0.375	0.423	0.398	0.187	0.167	0.177
123	√		rs2831700	21	29679687	SNP*for*ID	0.0536	0.496	0.465	0.480	0.377	0.394	0.386	0.186	0.179	0.182
124	√		rs2833736	21	33582722	Pakstis et al	0.0072	0.499	0.499	0.491	0.376	0.376	0.380	0.187	0.187	0.185
125			rs914165	21	42415929	SNP*for*ID	0.0253	0.434	0.474	0.500	0.415	0.389	0.375	0.170	0.181	0.187
126	√	√	rs221956	21	43606997	Pakstis et al	0.0018	0.490	0.460	0.483	0.380	0.397	0.384	0.185	0.177	0.183
127			rs5746846	22	19920646	Pakstis et al	0.0058	0.429	0.482	0.444	0.418	0.385	0.408	0.169	0.183	0.173
128			rs2073383	22	23802171	Pakstis et al	0.0011	0.476	0.439	0.474	0.388	0.411	0.389	0.181	0.171	0.181
129			rs733164	22	27816784	SNP*for*ID	0.0701	0.202	0.418	0.208	0.658	0.426	0.648	0.091	0.165	0.093
130	√	√	rs987640	22	33559508	Pakstis et al	0.0239	0.498	0.499	0.463	0.376	0.375	0.396	0.187	0.187	0.178
131			rs2040411	22	47836412	SNP*for*ID	0.0642	0.296	0.483	0.361	0.540	0.384	0.473	0.126	0.183	0.148
132			rs1028528	22	48362290	SNP*for*ID	0.0053	0.472	0.413	0.431	0.390	0.430	0.417	0.180	0.164	0.169

*Fifty-eight unlinked IISNPs (r^2<0.094) with H_e>0.450 and F_{st} values from 0.0002 to 0.0536.

†One recommended marker (rs2920816 by Pakstis et al) for which the analysis in MassARRAY design failed was eliminated.

‡F_{st} value was calculated with the Han, Uyghur, and Tibetan populations.
