# Supplementary material for: Validation of 58 autosomal individual identification SNPs in three Chinese populations
Source: Croat Med J. 2014 Feb;55(1):10–3. doi: 10.3325/cmj.2014.55.10 (PMC3944413; doi:10.3325/cmj.2014.55.10)
Supplement: Supplementary Table 2 [file CroatMedJ_55_s002.pdf]

Table S2. Allele frequency and pair-wise comparison of different populations

No.	dbSNP rs#	Chromesome	Nucleotide Position Map Build 36.2	A allele (B allele)	Allele frequency			δ*		
					Han	Uyghur	Tibetan	Han/Uyghur	Han/Tibetan	Uyghur/Tibetan
1	rs1490413	1	4367323	A (G)	0.537	0.482	0.623	0.055	0.086	0.141
2	rs7520386	1	14155402	A (G)	0.817	0.750	0.796	0.067	0.021	0.046
3	rs4847034	1	105717631	A (G)	0.523	0.307	0.468	0.216	0.055	0.161
4	rs560681	1	160786670	A (G)	0.676	0.555	0.495	0.121	0.181	0.060
5	rs1294331	1	233448413	A (G)	0.278	0.386	0.358	0.108	0.080	0.028
6	rs10495407	1	238439308	A (G)	0.349	0.345	0.355	0.004	0.006	0.010
7	rs891700	1	239881926	A (G)	0.477	0.468	0.500	0.009	0.023	0.032
8	rs1413212	1	242806797	A (G)	0.560	0.450	0.550	0.110	0.010	0.100
9	rs876724	2	114974	C (T)	0.550	0.665	0.705	0.115	0.155	0.040
10	rs1109037	2	10085722	A (G)	0.399	0.418	0.500	0.019	0.101	0.082
11	rs993934	2	124109213	C (T)	0.431	0.495	0.468	0.064	0.037	0.027
12	rs12997453	2	182413259	A (G)	0.326	0.289	0.335	0.037	0.009	0.046
13	rs907100	2	239563579	C (G)	0.583	0.500	0.555	0.083	0.028	0.055
14	rs1357617	3	961782	A (T)	0.844	0.816	0.921	0.028	0.077	0.105
15	rs4364205	3	32417644	G (T)	0.610	0.630	0.625	0.020	0.015	0.005
16	rs9866013	3	59488340	C (T)	0.239	0.350	0.300	0.111	0.061	0.050
17	rs2399332	3	110301126	A (C)	0.243	0.370	0.292	0.127	0.049	0.078
18	rs1872575	3	113804979	C (T)	0.477	0.500	0.495	0.023	0.018	0.005
19	rs1355366	3	190806108	A (G)	0.875	0.614	0.832	0.261	0.043	0.218
20	rs6444724	3	193207380	C (T)	0.330	0.350	0.255	0.020	0.075	0.095
21	rs2046361	4	10969059	A (T)	0.610	0.630	0.615	0.020	0.005	0.015

22	rs279844	4	46329655	A (T)	0.421	0.568	0.486	0.147	0.065	0.082
23	rs13134862	4	76425896	A (G)	0.440	0.459	0.536	0.019	0.096	0.077
24	rs1554472	4	157489906	C (T)	0.514	0.609	0.605	0.095	0.091	0.004
25	rs6811238	4	169663615	G (T)	0.633	0.545	0.695	0.088	0.062	0.150
26	rs1979255	4	190318080	C (G)	0.593	0.614	0.505	0.021	0.088	0.109
27	rs717302	5	2879395	A (G)	0.908	0.695	0.909	0.213	0.001	0.214
28	rs159606	5	17374898	A (G)	0.481	0.414	0.399	0.067	0.082	0.015
29	rs13182883	5	136633338	A (G)	0.500	0.366	0.417	0.134	0.083	0.051
30	rs7704770	5	159487953	A (G)	0.597	0.626	0.722	0.029	0.125	0.096
31	rs315791	5	169735920	A (C)	0.248	0.477	0.245	0.229	0.003	0.232
32	rs251934	5	174778678	C (T)	0.151	0.229	0.073	0.078	0.078	0.156
33	rs338882	5	178690725	C (T)	0.393	0.454	0.377	0.061	0.016	0.077
34	rs1029047	6	1135939	A (T)	0.367	0.349	0.349	0.018	0.018	0.000
35	rs13218440	6	12059954	A (G)	0.436	0.364	0.236	0.072	0.200	0.128
36	rs2811231	6	55155704	A (C)	0.423	0.593	0.382	0.170	0.041	0.211
37	rs1336071	6	94537255	A (G)	0.449	0.472	0.397	0.023	0.052	0.075
38	rs1478829	6	120560694	A (T)	0.569	0.629	0.620	0.060	0.051	0.009
39	rs1358856	6	123894978	A (C)	0.519	0.476	0.541	0.043	0.022	0.065
40	rs2503107	6	127463376	A (C)	0.602	0.528	0.514	0.074	0.088	0.014
41	rs2272998	6	148761456	C (G)	0.463	0.444	0.454	0.019	0.009	0.010
42	rs214955	6	152697706	A (G)	0.593	0.546	0.659	0.047	0.066	0.113
43	rs727811	6	165045334	A (C)	0.862	0.889	0.871	0.027	0.009	0.018
44	rs6955448	7	4310365	C (T)	0.718	0.641	0.670	0.077	0.048	0.029
45	rs917118	7	4457003	C (T)	0.661	0.651	0.750	0.010	0.089	0.099
46	rs1019029	7	13894276	C (T)	0.582	0.435	0.678	0.147	0.096	0.243
47	rs321198	7	137029838	C (T)	0.532	0.532	0.630	0.000	0.098	0.098
48	rs737681	7	155990813	C (T)	0.870	0.731	0.750	0.139	0.120	0.019

49	rs763869	8	1375610	C (T)	0.367	0.264	0.191	0.103	0.176	0.073
50	rs10092491	8	28411072	C (T)	0.665	0.670	0.752	0.005	0.087	0.082
51	rs4288409	8	136839229	A (C)	0.391	0.343	0.385	0.048	0.006	0.042
52	rs2056277	8	139399116	C (T)	0.884	0.849	0.830	0.035	0.054	0.019
53	rs4606077	8	144656754	C (T)	0.761	0.655	0.777	0.106	0.016	0.122
54	rs1015250	9	1823774	C (G)	0.564	0.382	0.455	0.182	0.109	0.073
55	rs2270529	9	14747133	C (T)	0.220	0.321	0.177	0.101	0.043	0.144
56	rs7041158	9	27985938	C (T)	0.623	0.591	0.632	0.032	0.009	0.041
57	rs1463729	9	126881448	A (G)	0.435	0.445	0.373	0.010	0.062	0.072
58	rs1360288	9	128968063	C (T)	0.661	0.618	0.714	0.043	0.053	0.096
59	rs10776839	9	137417308	G (T)	0.601	0.577	0.679	0.024	0.078	0.102
60	rs826472	10	2406631	C (T)	0.832	0.771	0.755	0.061	0.077	0.016
61	rs735155	10	3374178	A (G)	0.810	0.605	0.714	0.205	0.096	0.109
62	rs3780962	10	17193346	C (T)	0.394	0.550	0.441	0.156	0.047	0.109
63	rs1410059	10	97172595	C (T)	0.667	0.559	0.514	0.108	0.153	0.045
64	rs740598	10	118506899	A (G)	0.615	0.623	0.691	0.008	0.076	0.068
65	rs964681	10	132698419	C (T)	0.338	0.327	0.409	0.011	0.071	0.082
66	rs10500617	11	5099393	A (T)	0.611	0.683	0.591	0.072	0.020	0.092
67	rs1498553	11	5709028	C (T)	0.486	0.435	0.472	0.051	0.014	0.037
68	rs901398	11	11096221	C (T)	0.259	0.241	0.300	0.018	0.041	0.059
69	rs6591147	11	105912984	C (T)	0.550	0.564	0.605	0.014	0.055	0.041
70	rs10488710	11	115207176	C (G)	0.711	0.664	0.745	0.047	0.034	0.081
71	rs590162	11	122195989	A (G)	0.674	0.473	0.673	0.201	0.001	0.200
72	rs2076848	11	134667546	A (T)	0.338	0.505	0.332	0.167	0.006	0.173
73	rs2107612	12	888320	A (G)	0.876	0.786	0.918	0.090	0.042	0.132
74	rs2255301	12	6909442	C (T)	0.674	0.568	0.627	0.106	0.047	0.059
75	rs2269355	12	6945914	C (G)	0.450	0.459	0.427	0.009	0.023	0.032

76	rs2111980	12	106328254	A (G)	0.583	0.575	0.654	0.008	0.071	0.079
77	rs10773760	12	130761696	A (G)	0.667	0.605	0.614	0.062	0.053	0.009
78	rs1335873	13	20901724	A (T)	0.271	0.391	0.298	0.120	0.027	0.093
79	rs1886510	13	22374700	C (T)	0.855	0.688	0.991	0.167	0.136	0.303
80	rs1058083	13	100038233	A (G)	0.343	0.400	0.409	0.057	0.066	0.009
81	rs354439	13	106938411	A (T)	0.435	0.473	0.472	0.038	0.037	0.001
82	rs1454361	14	25850832	A (T)	0.459	0.432	0.359	0.027	0.100	0.073
83	rs722290	14	53216723	C (G)	0.468	0.459	0.432	0.009	0.036	0.027
84	rs873196	14	98845531	C (T)	0.156	0.277	0.118	0.121	0.038	0.159
85	rs4530059	14	104769149	A (G)	0.284	0.274	0.212	0.010	0.072	0.062
86	rs1821380	15	39313402	C (G)	0.315	0.341	0.468	0.026	0.153	0.127
87	rs8037429	15	53616909	C (T)	0.440	0.505	0.408	0.065	0.032	0.097
88	rs1528460	15	55210705	C (T)	0.413	0.376	0.569	0.037	0.156	0.193
89	rs729172	16	5606197	A (C)	0.114	0.314	0.170	0.200	0.056	0.144
90	rs2342747	16	5868700	A (G)	0.307	0.194	0.301	0.113	0.006	0.107
91	rs7205345	16	7520254	C (G)	0.650	0.542	0.639	0.108	0.011	0.097
92	rs430046	16	78017051	C (T)	0.634	0.634	0.593	0.000	0.041	0.041
93	rs1382387	16	80106361	G (T)	0.329	0.280	0.295	0.049	0.034	0.015
94	rs9905977	17	2919393	A (G)	0.384	0.321	0.455	0.063	0.071	0.134
95	rs740910	17	5706623	A (G)	0.922	0.836	0.955	0.086	0.033	0.119
96	rs4796362	17	6811529	A (G)	0.528	0.459	0.505	0.069	0.023	0.046
97	rs8070085	17	41341984	A (G)	0.697	0.686	0.573	0.011	0.124	0.113
98	rs1004357	17	41691526	A (G)	0.583	0.656	0.586	0.073	0.003	0.070
99	rs1027895	17	46510697	A (G)	0.619	0.661	0.709	0.042	0.090	0.048
100	rs938283	17	77468498	C (T)	0.144	0.118	0.055	0.026	0.089	0.063
101	rs8078417	17	80461935	C (T)	0.639	0.741	0.686	0.102	0.047	0.055
102	rs2291395	17	80526139	A (G)	0.596	0.536	0.582	0.060	0.014	0.046

103	rs3744163	17	80739859	C (G)	0.546	0.574	0.444	0.028	0.102	0.130
104	rs9546538	17	84456735	C (T)	0.375	0.314	0.427	0.061	0.052	0.113
105	rs1493232	18	1127986	A (C)	0.269	0.593	0.273	0.324	0.004	0.320
106	rs9951171	18	9749879	A (G)	0.407	0.481	0.514	0.074	0.107	0.033
107	rs7229946	18	22739001	A (G)	0.366	0.408	0.405	0.042	0.039	0.003
108	rs985492	18	29311034	C (T)	0.450	0.409	0.455	0.041	0.005	0.046
109	rs521861	18	47371014	C (G)	0.565	0.500	0.588	0.065	0.023	0.088
110	rs1736442	18	55225777	A (G)	0.358	0.373	0.366	0.015	0.008	0.007
111	rs1024116	18	75432386	A (G)	0.133	0.323	0.136	0.190	0.003	0.187
112	rs719366	19	28463337	C (T)	0.217	0.386	0.318	0.169	0.101	0.068
113	rs576261	19	39559807	A (C)	0.421	0.455	0.500	0.034	0.079	0.045
114	rs1031825	20	4447483	A (C)	0.321	0.345	0.450	0.024	0.129	0.105
115	rs445251	20	15124933	C (G)	0.330	0.359	0.300	0.029	0.030	0.059
116	rs12480506	20	16241416	A (G)	0.632	0.743	0.574	0.111	0.058	0.169
117	rs2567608	20	23017082	A (G)	0.398	0.410	0.403	0.012	0.005	0.007
118	rs1005533	20	39487110	A (G)	0.329	0.343	0.366	0.014	0.037	0.023
119	rs1523537	20	51296162	C (T)	0.380	0.491	0.317	0.111	0.063	0.174
120	rs722098	21	16685598	A (G)	0.463	0.601	0.382	0.138	0.081	0.219
121	rs464663	21	28023370	C (T)	0.734	0.579	0.634	0.155	0.100	0.055
122	rs2830795	21	28608163	A (G)	0.486	0.697	0.642	0.211	0.156	0.055
123	rs2831700	21	29679687	A (G)	0.454	0.632	0.400	0.178	0.054	0.232
124	rs2833736	21	33582722	A (G)	0.477	0.473	0.568	0.004	0.091	0.095
125	rs914165	21	42415929	A (G)	0.318	0.385	0.486	0.067	0.168	0.101
126	rs221956	21	43606997	C (T)	0.569	0.641	0.591	0.072	0.022	0.050
127	rs5746846	22	19920646	C (G)	0.312	0.404	0.332	0.092	0.020	0.072
128	rs2073383	22	23802171	C (T)	0.610	0.674	0.614	0.064	0.004	0.060
129	rs733164	22	27816784	A (G)	0.114	0.298	0.118	0.184	0.004	0.180

130	rs987640	22	33559508	A (T)	0.472	0.518	0.636	0.046	0.164	0.118
131	rs2040411	22	47836412	A (G)	0.181	0.407	0.236	0.226	0.055	0.171
132	rs1028528	22	48362290	A (G)	0.619	0.709	0.686	0.090	0.067	0.023

*δ value indicated allele frequency difference of pairwise comparison.
